# Enhancing salinity tolerance in potato (*Solanum tuberosum* L.) using foliar-applied branched-chain amino acids

**DOI:** 10.1038/s41598-026-60493-y

**Published:** 2026-07-07

**Authors:** Mohamed A. I. Khalefa, Nabil Ahmed Younes, Ahmed Fathy Yousef

**Affiliations:** Horticulture Department, Agriculture College, Al-Azhar University (Assiut Branch), Assiut, 71524 Egypt

**Keywords:** Bio-stimulant, Branched-chain amino acids, Foliar application, Growth and yield, Salinity stress, Physiological responses, Physiology, Plant sciences

## Abstract

Salinity stress is a major constraint to potato production in sandy soils under arid environments. This study evaluated the effectiveness of foliar-applied branched-chain amino acids (BCAAs)—valine, leucine, and isoleucine—applied individually or in combinations to enhance salinity tolerance of potato (*Solanum tuberosum* L. cv. Kara) during the 2023 and 2024 growing seasons in Wadi El Natrun. A split-plot randomized complete block design with three replicates was used, where three salinity levels (0, 1500, and 3000 ppm NaCl) were assigned to main plots, while eight BCAA treatments were allocated to subplots: control (water spray), valine (2 g L⁻¹), leucine (2 g L⁻¹), isoleucine (2 g L⁻¹), valine + leucine (1 g L⁻¹ each), valine + isoleucine (1 g L⁻¹ each), leucine + isoleucine (1 g L⁻¹ each), and valine + leucine + isoleucine (0.75 g L⁻¹ each). Salinity significantly reduced vegetative growth, tuber number, and total yield, particularly at 3000 ppm NaCl. Foliar BCAA application mitigated these adverse effects. Under moderate salinity, isoleucine and dual combinations increased yield up to 45.22 tons ha⁻¹ versus 29.35 tons ha⁻¹ in untreated plants. Under severe salinity, the combined treatment (V + L+I) produced the highest yield (42.05 tons ha⁻¹). Enhanced proline accumulation, protein content, and antioxidant enzyme activity indicated improved stress tolerance. BCAAs represent an effective strategy for sustaining potato productivity in salt-affected soils.

## Introduction

Salinity stress is one of the most serious abiotic factors limiting agricultural productivity worldwide, particularly in arid and semi-arid regions such as Egypt^1,2^. High salt concentrations in soil or irrigation water adversely affect plant growth, physiological processes, and yield by inducing osmotic stress, ionic imbalance, and oxidative damage^3,4^. Potato (*Solanum tuberosum* L.), as one of the most important food crops globally, is considered moderately sensitive to salinity, making its productivity highly vulnerable under saline conditions^5,6^. Global potato production exceeds 370 million tons annually, with an average productivity of approximately 21–22 tons ha⁻¹, while commercial production systems often achieve yields above 40 tons ha⁻¹ under optimal management conditions^7^. In Egypt, potato is an important cash and export crop cultivated extensively in both old and newly reclaimed lands, with national production reaching approximately 8.08 million tons in 2024^8^. However, potato is considered moderately sensitive to salinity, and yield losses may become substantial when soil salinity exceeds threshold levels, making its productivity highly vulnerable in salt-affected sandy soils^9^.

To mitigate the negative effects of salinity, various agronomic and biochemical strategies have been explored^10,11^. Salinity stress primarily arises from the excessive accumulation of soluble salts in the soil, which creates both osmotic stress and ion toxicity in plants^12^. These conditions disrupt water uptake, cause ionic imbalance (particularly Na⁺ and Cl⁻ accumulation), and lead to oxidative stress. Consequently, plant morphophysiological processes such as seed germination, root and shoot growth, photosynthetic efficiency, stomatal conductance, and chlorophyll content are significantly reduced^13,14^. Ultimately, these alterations result in lower biomass production and substantial yield losses in many crop species^14^. Among these, the exogenous application of amino acids has gained increasing attention due to their role in plant metabolism and stress tolerance^2,15,16^. Amino acids not only serve as building blocks of proteins but also function as osmoprotectants, antioxidants, and signaling molecules that regulate plant responses under stress conditions^17^.

Branched-chain amino acids (BCAAs), including valine, leucine, and isoleucine, are particularly important due to their involvement in stress-related metabolic pathways^18–20^. These amino acids contribute to osmotic adjustment, stabilization of cellular structures, and enhancement of antioxidant defense systems^15,21^. Moreover, they may improve nitrogen metabolism and energy balance in plants exposed to adverse environmental conditions such as salinity stress^22^. BCAAs contribute to salinity tolerance through multiple interconnected mechanisms. They are involved in osmotic adjustment by contributing to compatible solute pools, thereby helping maintain cellular water balance under high salt conditions^23^. In addition, they enhance the antioxidant defense system by stimulating the activity of key enzymes such as superoxide dismutase (SOD), catalase (CAT), and peroxidase (POD), which reduces reactive oxygen species (ROS)-induced oxidative damage^24^. Furthermore, BCAAs play an important role in maintaining nitrogen metabolism and energy homeostasis, supporting protein synthesis and metabolic recovery under stress conditions^22,25^. BCAAs may also contribute to membrane stabilization and act as key regulators of stress-responsive signaling pathways and gene expression, as their accumulation—controlled by enzymes such as BCATs and DHAD in the biosynthetic pathway—has been associated with enhanced tolerance to drought and salinity stresses in plants^26^.

Despite the recognized importance of amino acids, limited studies have investigated the combined and individual effects of BCAAs on salinity tolerance in potato plants, especially under field conditions. Understanding their potential role could provide a practical and sustainable approach to improving crop performance in saline soils. We hypothesize that exogenous foliar application of branched-chain amino acids (valine, leucine, and isoleucine), either individually or in combination, enhances salinity tolerance in potato plants by improving growth performance, physiological efficiency, and biochemical stress responses, ultimately leading to improved yield under salt stress conditions. Therefore, the present study aimed to evaluate the effectiveness of foliar application of valine, leucine, and isoleucine—individually and in combination—in enhancing salinity tolerance of potato plants grown under different levels of salt stress. The study focused on assessing their impact on growth, yield, and physiological and biochemical parameters.

## Materials and methods

### Experimental site

This study was carried out during the summer growing seasons of 2023 and 2024 at a private farm in Wadi El-Natroun City, Beheira Governorate, Egypt (30° 27’ 20’’ N, 30° 10’ 20’’ E). The experimental site featured sandy soil, with detailed soil characteristics provided in Table 1.

**Table 1:** The physical and chemical characteristics of the farm experimental soil.

Characteristic		Value	Characteristic	Value
O.M.%		0.82	Mg^+ 2^	0.172
CaCO_3_%		1.008	Na ^+^	0.125
Sand%	Coarse sand	57.63	K^+^	0.013
	Fine Sand	38.23		
Silt %		2.05	SO4^− 2^	0.174
Clay%		2.09	Ca^+ 2^	0.138
Texture class		Sandy	Available (mg/kg)	
pH		7.62	N	15.11
EC (dS/m)		2.33		
Soluble ions (me/L)			P	7.2
CO_3_		---		
HCO_3_		0.76	K	75.80
**Cl**		0.188		

### Experimental plant and design

This study was conducted during the summer growing seasons of 2023 and 2024 at a private farm in Wadi El Natrun, Beheira Governorate, Egypt (30°25’N, 30°19’E), utilizing sandy desert soil conditions to evaluate the efficacy of foliar-applied branched-chain amino acids (valine, leucine, and isoleucine) in enhancing salinity tolerance of potato (*Solanum tuberosum* L. cv. Kara) plants. The investigation specifically examined the potential of individual and combined amino acid treatments to mitigate salt stress effects on potato growth, yield, and physiological parameters under three salinity irrigation regimes (0, 1500, and 3000 ppm NaCl). The study utilized the commercial potato variety Kara in a randomized complete block design (RCBD) with three replicates, employing a split-plot arrangement. Main plots were assigned to salt stress treatments (Factor A: 0 [control water without NaCl], 1500, and 3000 ppm NaCl irrigation), while subplots were allocated to foliar branched-chain amino acids (BCAAs) applications (Factor B: 8 treatments, including Control (spray water); Valine (2 g L⁻¹); Leucine (2 g L⁻¹); Isoleucine (2 g L⁻¹); Valine (1 g L⁻¹)+ Leucine (1 g L⁻¹); Valine (1 g L⁻¹)+Isoleucine (1 g L⁻¹); Leucine (1 g L⁻¹)+ Isoleucine (1 g L⁻¹); Valine (0.75 g L⁻¹)+ Leucine (0.75 g L⁻¹)+ Isoleucine (0.75 g L⁻¹) as shown in Table 2. These concentrations were selected based on a preliminary experiment in which potato plants were treated with BCAA concentrations ranging from 0.5 to 2.5 g L⁻¹ under moderate salinity (1500 ppm NaCl); the concentrations yielding the highest vegetative growth and tuber yield were chosen for the main study. Climatic data for the experimental periods, including average, minimum, and maximum air temperature, relative humidity, and rainfall, were obtained from Central Laboratory for Agricultural Climate (Giza, Egypt) and are presented in Table  2 and 3.

**Table 2:** Treatments of the experiment and code used in the thesis.

Factor A	Factor B		Code
0 NaCl [Control] (S0)	Control (C) (spray water)		S0C
	Valine (2 g L⁻¹) - (V)		S0V
	Leucine (2 g L⁻¹) - (L)		S0L
	Isoleucine (2 g L⁻¹) - (I)		S0I
	Valine (1 g L⁻¹) + Leucine (1 g L⁻¹) - (VL)		S0VL
	Valine (1 g L⁻¹) +Isoleucine (1 g L⁻¹) - (VI)		S0VI
	Leucine (1 g L⁻¹) + Isoleucine (1 g L⁻¹) - (LI)		S0LI
	Valine (0.75 g L⁻¹) + Leucine (0.75 g L⁻¹) + Isoleucine (0.75 g L⁻¹) - (VLI)		S0VLI
1500 ppm NaCl (S1.5)	Control (C) (spray water)		S1.5 C
	Valine (2 g L⁻¹) - (V)		S1.5 V
	Leucine (2 g L⁻¹) - (L)		S1.5 L
	Isoleucine (2 g L⁻¹) - (I)		S1.5I
	Valine (1 g L⁻¹) + Leucine (1 g L⁻¹) - (VL)		S1.5VL
	Valine (1 g L⁻¹) +Isoleucine (1 g L⁻¹) - (VI)		S1.5VI
	Leucine (1 g L⁻¹) + Isoleucine (1 g L⁻¹) - (LI)		S1.5LI
	Valine (0.75 g L⁻¹) + Leucine (0.75 g L⁻¹) + Isoleucine (0.75 g L⁻¹) - (VLI)		S1.5VLI
3000 ppm NaCl (S3)	Control (C) (spray water)		S3C
	Valine (2 g L⁻¹) - (V)		S3V
	Leucine (2 g L⁻¹) - (L)		S3L
	Isoleucine (2 g L⁻¹) - (I)		S3I
	Valine (1 g L⁻¹) + Leucine (1 g L⁻¹) - (VL)		S3VL
	Valine (1 g L⁻¹) +Isoleucine (1 g L⁻¹) - (VI)		S3VI
	Leucine (1 g L⁻¹) + Isoleucine (1 g L⁻¹) - (LI)		S3LI
	Valine (0.75 g L⁻¹) + Leucine (0.75 g L⁻¹) + Isoleucine (0.75 g L⁻¹) - (VLI)		S3VLI

**Table 3:** Climatic conditions during the experimental periods (January–May) of the 2023 and 2024 growing seasons at Wadi El Natrun, Egypt.

	Month	T max (°C)	T min (°C)	RH (%)	Rainfall (mm)
2023	January	18.5	8.4	60	10
	February	20.0	8.6	55	9
	March	23.4	10.1	52	6
	April	27.2	12.5	50	2
	May	30.8	15.7	51	1
2024	January	17.5	8.0	61	11
	February	19.5	8.3	56	10
	March	23.0	9.8	54	7
	April	27.0	12.2	52	2
	May	30.3	15.5	54	1

### Field preparation and cultivation

Prior to planting, the sandy soil was leached for two consecutive days using drip irrigation to remove accumulated salts from the root zone. A leaching fraction of approximately 15–20% was maintained based on the ratio between drainage water and applied irrigation water. Soil electrical conductivity before planting was 2.33 dS m⁻¹; however, soil solution EC after leaching was not measured. During land preparation, 47.6 m³ ha⁻¹ of animal organic manure and 595 kg ha⁻¹ of mono superphosphate fertilizer (15.5% *P* ≈ 92.2 kg P₂O₅ ha⁻¹) were incorporated into the soil.

The experimental field was divided into experimental plots. Each plot measured 2.40 m in width (three rows, each 80 cm wide) × 3.0 m in length, with each treatment replicated three times. Buffer zones were maintained between plots to prevent interference among treatments. Each experimental plot contained 18 plants per replicate.

During the growing season, fertilizers were applied through the irrigation system as follows: 71.4 L ha⁻¹ phosphoric acid (H₃PO₄, 85%), 833 kg ha⁻¹ ammonium fertilizer (33.5% N), 214.2 kg ha⁻¹ calcium nitrate [Ca(NO₃)₂], 119 kg ha⁻¹ magnesium sulfate (MgSO₄·7 H₂O), and 273.7 kg ha⁻¹ potassium sulfate (K₂SO₄, 48–50% K₂O). In addition, 4.76 kg ha⁻¹ of a micronutrient mixture (Fe, Zn, Mn, Cu, and B) was applied as foliar sprays in three separate applications.

### Planting and irrigation

Tubers (approximately 35 g) were cut, air-dried for one week, and manually planted on 22 January during both the 2023 and 2024 growing seasons. Tubers were planted at a spacing of 15 cm between hills and 80 cm between rows.

Irrigation was applied through a drip irrigation system using Nile River water and was scheduled according to standard local potato production practices for sandy soils. A total seasonal irrigation volume of approximately 4500 m³ feddan⁻¹ was applied uniformly across all treatments. Irrigation was performed every three days during the crop establishment stage and two days during tuber initiation and bulking stages, depending on weather conditions and soil moisture requirements. The baseline salinity of the Nile River irrigation water was 300 ppm, which was used as the control treatment. One week after planting, salinity treatments were imposed by supplementing the irrigation water with NaCl to achieve two additional salinity levels. Accordingly, the experimental treatments consisted of 300 ppm (control), 1800 ppm (300 ppm baseline water salinity + 1500 ppm added NaCl), and 3300 ppm (300 ppm baseline water salinity + 3000 ppm added NaCl) as presented in Table 4. Salinity levels were expressed as NaCl concentration because treatments were prepared by direct salt addition rather than being classified according to ECw values.

**Table 4:** Irrigation water characteristics under different salinity treatments.

Salinity treatment	Base irrigation water salinity (ppm)	Added NaCl (ppm)	Final irrigation water salinity (ppm)	Seasonal water applied (m³/feddan)
Control (S0)	300	0	300	4500
Moderate salinity (S1.5)	300	1500	1800	4500
High salinity (S3)	300	3000	3300	4500

The amino acid compounds used in this study were completely soluble in water and did not require additional solvents. The solutions were prepared using Nile River water at normal ambient temperature. Amino acid treatments were applied as foliar sprays during the vegetative growth stage using a hand sprayer until full leaf coverage and saturation were achieved. Applications were performed four times during each growing season at 42, 52, 65, and 75 days after planting.

### Data recorded

#### Vegetative growth and yield parameters

Plant and tuber samples were collected to evaluate growth and yield indicators. Plant height (cm), stem diameter (mm), and number of stems per plant were recorded as vegetative indicators, while tuber yield (tons/ feddan) and number of tubers per plant were assessed from a representative 3.5-meter row segment harvested from the central rows of each plot. Additionally, randomly selected tubers were laboratory-graded by diameter into three size categories: large (> 6.5 cm), medium (5.0–6.5 cm), and small (2.5–5.0 cm) to determine size distribution patterns under different treatment conditions.

### Chemical composition of potato leaves

#### Determination of proline content

Proline content in dried potato leaves was estimated using the acid-ninhydrin method of Bates, et al. ^27^. Briefly, 0.1 g of finely ground leaf tissue was homogenized in 5 mL of 3% sulfosalicylic acid, boiled for 10 min, and centrifuged at 10,000 rpm for 10 min. The supernatant (1 mL) was reacted with 1 mL each of acid-ninhydrin reagent (1.25 g ninhydrin in 30 mL glacial acetic acid and 20 mL 6 M phosphoric acid) and glacial acetic acid at 100 °C for 30 min. After cooling, the chromophore was extracted with 2 mL toluene, and absorbance was measured at 520 nm using a spectrophotometer. Proline concentration was determined using a standard curve of L-proline (0–100 µg mL^-1^ and expressed as µg proline per gram dry weight (µg g⁻¹ DW). Proline content equation as following:

$${\mathrm{Proline}}~\left( {\mu {\text{g g}}^{{ - {\mathrm{1}}}} ~{\mathrm{DW}}} \right) = \frac{{\left( {\mu g{\text{proline from curve}} \times {\text{Extraction volume}}} \right)}}{{{\text{Sample weight}}\left( {\mathrm{g}} \right)}}$$

#### Determination of carbohydrate content

Total carbohydrates were quantified using the anthrone method as described by Sadasivam^28^. Briefly, 0.1 g of plant powder was hydrolyzed by boiling in 5 mL of 2.5 N HCl (Sigma-Aldrich, Egypt) for 3 h. After cooling to room temperature (27 °C), sodium carbonate (Sigma-Aldrich, Egypt) was added gradually until effervescence ceased. The resulting mixture was then made up to 100 mL with distilled water in a volumetric flask and centrifuged at 3000 rpm for 10 min. An aliquot (0.1 mL) of the supernatant was diluted to 1 mL with distilled water. Subsequently, 4 mL of pre-chilled anthrone reagent (BBI Life Sciences, China) was added, and the mixture was incubated in a boiling water bath for 8 min. After cooling, the developed green color was measured spectrophotometrically at 630 nm using a UV–Vis dual-beam spectrophotometer (UVS-2700, LABOMED Inc., USA). Carbohydrate concentration was determined using a standard calibration curve prepared with glucose. Carbohydrates content equation as following:

$${\text{Carbohydrates }}\left( {{\text{mg g}}^{{ - 1}} } \right) = ~~\frac{{\left( {{\mathrm{C}} \times {\mathrm{V~}} \times {\mathrm{DF}}} \right)}}{{\mathrm{W}}}$$

Where: C = concentration obtained from the standard curve (mg mL^− 1^); V = total volume of extract (mL); W = weight of sample (g); DF= dilution factor.

#### Determination of protein content

Protein content in dried potato leaves was determined using the Bradford method Bradford^29^. Leaf powder (0.1 g) was extracted with 5 mL of 50 mM Tris-HCl buffer (pH 7.5, 1% SDS) at 60 °C for 30 min. After centrifugation (10,000 rpm, 15 min), the supernatant was reacted with Bradford reagent (100 µL sample + 5 mL reagent), and absorbance was measured at 520 nm. Protein concentration was calculated using a BSA standard curve (0–1000 µg mL^−1^ and expressed as mg per g dry weight (DW). Protein content equation as following:

$${\mathrm{Protein}}~\left( {{\text{mg g}}^{{ - 1}} {\mathrm{DW}}} \right) = ~~\frac{{\left( {\mu g{\text{protein from curve}} \times {\text{Extraction volume}}\left( {{\mathrm{mL}}} \right)} \right)}}{{{\text{Sample weight}}\left( {\mathrm{g}} \right) \times 1000}}$$

#### Determination of total soluble sugars content

Total soluble sugars in dried potato leaves were quantified using the anthrone method Ludwig and Goldberg^30^. Briefly, 0.1 g of finely ground tissue was extracted twice with 5 mL of 80% ethanol at 80 °C for 30 min, followed by centrifugation (10,000 rpm, 10 min) to collect the sugar-rich supernatant. The pooled extract was evaporated to dryness and reconstituted in 5 mL distilled water. A standard curve was prepared using glucose solutions (0–100 µg mL^− 1^), with 0.5 mL of each standard or sample reacted with 3 mL ice-cold anthrone reagent (0.2% in concentrated H₂SO₄) and heated at 100 °C for 10 min to develop color. After cooling, absorbance was measured at 625 nm. The sugar concentration was determined by interpolating sample absorbance against the standard curve (typically linear with *R² ≥ 0.98*) and expressed as mg sugar per g dry weight (DW), accounting for dilution factors. For starch analysis, the ethanol-insoluble residue was hydrolyzed with perchloric acid and similarly assayed. Total soluble sugars equation as following:

$${\mathrm{Sugar}}~(\mu {\text{g g}}^{{ - {\mathrm{1}}}} {\mathrm{DW}}) =\frac{{\left[ {\left( {A~{\mathrm{sample}} - {\text{}}A~{\mathrm{blank}}} \right)/{\mathrm{Slope}}} \right]}}{{{\mathrm{Slope}}\left] { \times } \right[V{\mathrm{extract}}/{\text{Sample weight}}\left( {\mathrm{g}} \right)]}}$$

Variables Explained:

A sample: Absorbance of your plant sample at 625 nm.

A blank: Absorbance of the 0 µg/mL glucose standard (typically ~ 0.001).

Slope: From your standard curve equation (y = mx+cy = mx + c).

V extract: Total volume of sugar extract (e.g., 5 mL after resuspension).

Sample weight: Dry weight of tissue extracted (e.g., 0.1 g).

#### Determination of catalase (CAT) activity

Catalase (CAT; EC 1.11.1.6) activity was assessed by measuring the rate of hydrogen peroxide (H₂O₂) decomposition at 240 nm, following the protocols of Alici and Arabaci^31^ and Kadhum and Hadwan^32^, with slight modifications. Fresh leaf samples (0.5 g) were homogenized in 5 mL of ice-cold 50 mM sodium phosphate buffer (pH 7.5) supplemented with 1 mM EDTA, which serves to inhibit metal-catalyzed oxidation reactions. The homogenization was performed using a pre-chilled mortar and pestle. The resulting mixture was centrifuged at 10,000 × g for 20 min at 4 °C, and the clear supernatant was collected as the crude enzyme extract. The assay mixture consisted of 1.8 mL of 50 mM sodium phosphate buffer (pH 7.5) and 1.0 mL of 15 mM H₂O₂, which was prepared by diluting 0.2 mL of a 30% stock solution to 50 mL with the same buffer. The enzymatic reaction was initiated by adding 0.1 mL of the crude extract to the cuvette. The decrease in absorbance at 240 nm indicating H₂O₂ breakdown was monitored at 15-second intervals over 2 min using a UV-2100 spectrophotometer (Unico Co., China), maintained at a constant temperature of 25 °C. One unit of catalase activity was defined as the amount of enzyme necessary to decompose 1 µmol of H₂O₂ per minute under the specified conditions, based on an extinction coefficient of 39.4 M⁻¹ cm⁻¹ for H₂O₂. Enzyme activity was expressed as µmol H₂O₂ decomposed per minute per milligram of protein. Total protein content was determined using the Bradford assay^29^, with bovine serum albumin (BSA) serving as the standard. Catalase (CAT) activity equation as following:

$${\mathrm{CAT}}~{\mathrm{Activity}}~(\mu {\text{mol min}}^{{ - {\mathrm{1}}}} {\mathrm{mg}}^{{ - {\mathrm{1}}}} ~{\mathrm{protein}}){\text{ }} = \frac{{\left[ {{{\Delta }}A240/{\mathrm{min}} \times V{\mathrm{total}}} \right]}}{{\left[ {\times d \times V{\mathrm{enzyme}} \times {\mathrm{Protein}}\left( {{\mathrm{mg}}/{\mathrm{mL}}} \right)} \right]}}$$

Variables Explained:

ΔA/min: Absorbance change per minute (slope of linear phase).

V < sub> total</sub>: Total reaction volume (2.9 mL = 1.8 mL buffer + 1.0 mL H₂O₂ + 0.1 mL enzyme).

$\epsilon$ Extinction coefficient of H₂O₂ (39.4 M⁻¹ cm⁻¹ = 0.0394 mM⁻¹ cm⁻¹).

d: Pathlength (1 cm).

V < sub> enzyme</sub>: Volume of enzyme extract used (0.1 mL).

Protein: Concentration from Bradford assay (mg mL⁻¹)

#### Determination of peroxidase (POD) activity

Peroxidase (POD; EC 1.11.1.7) activity was determined by homogenizing 1.0 g fresh leaf tissue in 5 mL ice-cold 0.1 M sodium phosphate buffer (pH 6.5) containing 1% polyvinylpyrrolidone (PVP), followed by centrifugation at 10,000 × g for 20 min at 4 °C; the supernatant was adjusted to 3 mL with extraction buffer and combined with 3.0 mL of 0.05 M guaiacol and 1.0 mL of 0.1 M phosphate buffer (pH 6.5) in a quartz cuvette^31^. The peroxidase (POD) reaction was initiated by adding 0.1 mL of 0.8 M H₂O₂ to the reaction mixture containing 3.0 mL of 0.05 M guaiacol, 3.0 mL phosphate buffer (pH 6.5), and 0.1 mL enzyme extract. The formation of tetraguaiacol was monitored by measuring the increase in absorbance at 470 nm every 15 s for 3 min at 25 °C using a UV-2100 spectrophotometer (Unico Co.). Peroxidase activity was calculated using the following equation:

$${\mathrm{POD}}~{\mathrm{Activity}}~(\mu {\text{mol min}}^{{ - {\mathrm{1}}}} {\mathrm{g}}^{{ - {\mathrm{1}}}} ~{\mathrm{FW}}){\text{ }} = \frac{{\left[ {{{\Delta }}A470/{\mathrm{min}} \times V{\mathrm{total}}} \right]}}{{\left[ {~ \times d \times V{\mathrm{enzyme}} \times {\text{Fresh weight}}\left( {\mathrm{g}} \right)} \right]}}$$

Variables Explained:

ΔA/min: Absorbance change per minute (slope of linear phase).

V < sub> total</sub>: Total reaction volume (7.1 mL = 3.0 mL guaiacol + 3.0 mL buffer + 1.0 mL H₂O₂ + 0.1 mL enzyme).

ε: Extinction coefficient of tetraguaiacol (26.6 mM⁻¹ cm⁻¹).

d: Pathlength (1 cm).

V < sub> enzyme</sub>: Volume of enzyme extract used (0.1 mL).

Fresh weight (FW): Weight of leaf tissue extracted (1.0 g).

### Statistical analysis

The data obtained from these experiments were analyzed statistically using Statistix 8.1 software. A two-way Analysis of Variance (ANOVA) was conducted to evaluate the significance of different factors on the growth parameters and yield traits. To further investigate the differences among means that showed significant effects, Tukey HSD test at 5% was applied. This method allows for detailed comparisons between treatment means at a 95% confidence level^33^. For principal component analysis (PCA), the correlation matrix was subjected to PCA with Varimax rotation (Kaiser normalization) using XLSTAT (https://www.xlstat.com, New York, NY, USA).

## Results

### Vegetative characteristics and maturity

**Fig. 1:**
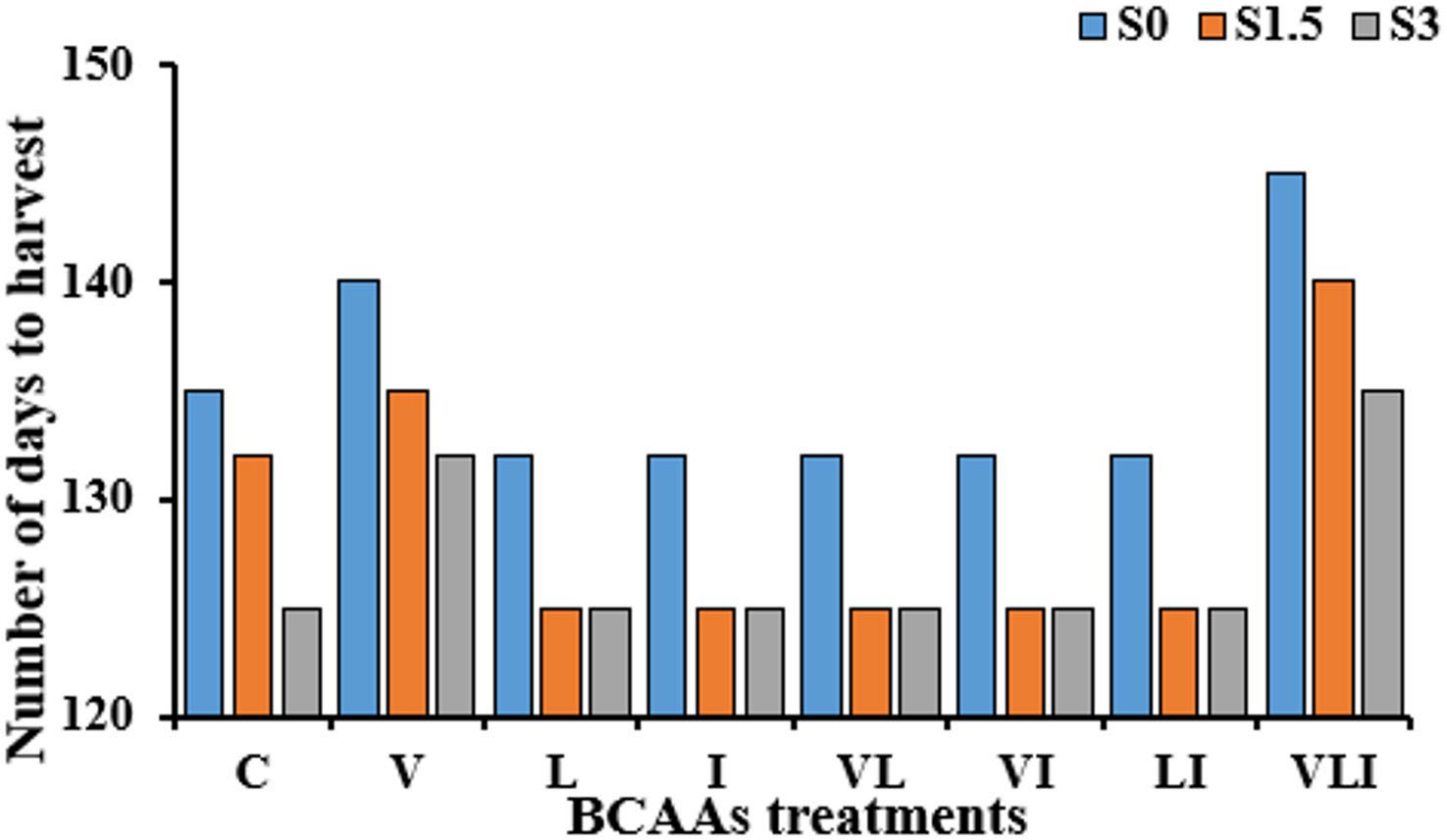
Number of days from planting to harvest. Where: S0 = 0 ppm NaCl [Control]; S1.5 = 1500 ppm NaCl; S3 = 3000 ppm NaCl; C= Control (spray water); V= Valine (2 g L⁻¹); L= Leucine (2 g L⁻¹); I= Isoleucine (2 g L⁻¹); VL= Valine (1 g L⁻¹)+ Leucine (1 g L⁻¹); VI= Valine (1 g L⁻¹)+Isoleucine (1 g L⁻¹); LI= Leucine (1 g L⁻¹)+ Isoleucine (1 g L⁻¹); VLI= Valine (0.75 g L⁻¹)+ Leucine (0.75 g L⁻¹)+ Isoleucine (0.75 g L⁻¹).

The application of branched-chain amino acids significantly influenced crop maturity duration (Fig. 1). Under non-saline conditions (S0), days to harvest ranged from 132 days (L, I, VL, VI, LI treatments) to 145 days (VLI treatment), compared to 135 days for the control. Under moderate salinity (S1.5, 1500 ppm NaCl), amino acid treatments generally accelerated maturity, with most treatments (L, I, VL, VI, LI) reaching harvest at 125 days compared to 132 days for the control—a reduction of 7 days. Under high salinity (S3, 3000 ppm NaCl), maturity time was further reduced to 125 days across most amino acid (L, I, VL, VI, and LI) treatments and the control, indicating accelerated senescence under severe stress. Notably, the triple combination (VLI) consistently extended the vegetative growth period across all salinity levels (145, 140, and 135 days for S0, S1.5, and S3, respectively).

Data presented in Table 5 showed that plant height was affected by both salinity levels and foliar application of branched-chain amino acids across both growing seasons. In general, plant height decreased progressively with increasing salinity, confirming the inhibitory effect of salt stress on vegetative growth. Under non-saline conditions (S0), amino acid treatments, particularly valine and leucine, produced the tallest plants in both seasons. Moderate salinity (S1.5) reduced plant height across all treatments, with relatively small differences among amino acid applications. The greatest reduction was observed under severe salinity (S3), where untreated plants consistently recorded the lowest plant height values. However, foliar application of BCAAs partially alleviated the negative effects of salinity, although their effectiveness varied depending on salinity level and season.

**Table 5:** Effect of foliar application of branched-chain amino acids on vegetative and yield parameters of potato plants under saline irrigation conditions during the 2023 and 2024 growing seasons.

Treatments		Plant height		Stem diameters		No. of Stem		Total No. of tubers		Total weight of tubers (Kg.)	
		2023	2024	2023	2024	2023	2024	2023	2024	2023	2024
S0	C	27.67^b−e^	18.83^fgh^	7.93 ^a−d^	5.53 ^a^	11.00 ^bcd^	10.00 ^cd^	37.00 ^a−d^	30 ^ab^	3.63 ^i^	3.27 ^def^
	V	38.93 ^a^	27.80 ^a^	8.83 ^a^	6.43 ^a^	10.00 ^d−g^	10.67 ^bc^	51.33^ab^	29.67^ab^	4.90 ^a^	3.63 ^b^
	L	35.3 ^a^	26.17 ^abc^	8.43 ^a^	7.03 ^a^	10.00 ^d−g^	11.00 ^abc^	46.00 ^a−d^	36.67^ab^	4.67 ^bc^	4.10 ^a^
	I	29.6 ^b^	25.27 ^a−e^	8.20 ^abc^	6.70 ^a^	10.67 ^b−e^	10.00 ^cd^	49.00 ^abc^	30.67^ab^	4.77 ^ab^	3.63 ^b^
	VL	24.17 ^c−f^	26.6 ^ab^	8.23 ^abc^	6.93 ^a^	10 ^d−g^	10.67 ^bc^	41.00 ^a−d^	33.67^ab^	4.33^efg^	4.00 ^a^
	VI	29.00 ^bc^	26.53 ^abc^	7.57 ^a−f^	6.47 ^a^	11.33 ^abc^	10.00 ^cd^	38.33 ^a−d^	29.67^ab^	4.40 ^def^	4.00 ^a^
	LI	29.93 ^b^	22.3 ^b−f^	8.40 ^ab^	5.90 ^a^	10.33 ^c−f^	10.00 ^cd^	33.33 ^a−d^	27 ^ab^	4.50 ^cde^	3.53 ^bc^
	VLI	24.27 ^c−f^	22.9 ^b−f^	6.97 ^c−g^	5.93 ^a^	10.00 ^d−g^	10.33 ^bcd^	33.33 ^a−d^	27.33^ab^	4.90 ^a^	3.90 ^a^
S1.5	C	18.1 ^h−k^	16.37 ^hij^	6.57 ^efg^	6.13 ^a^	11.67^ab^	11.00 ^abc^	32.00 ^a−d^	28.33^ab^	2.87 ^k^	2.47 ^ij^
	V	24.9 ^b−f^	25.57 ^a−d^	7.97 ^a−d^	7.57 ^a^	11.00 ^bcd^	10.00 ^cd^	38.33 ^a−d^	37.67 ^ab^	3.63 ^i^	3.63 ^b^
	L	25.47 ^b−f^	21.60 ^d−g^	7.60 ^a−f^	7.07 ^a^	10.67 ^b−e^	10.00 ^cd^	42.33 ^a−d^	34 ^ab^	3.97 ^h^	3.37 ^cde^
	I	23.37 ^d−g^	21.00 ^efg^	6.33 ^fg^	7.73 ^a^	12.33 ^a^	8.67 ^ef^	53.00 ^a^	33.33 ^ab^	4.23 ^fg^	3.63 ^b^
	VL	28.4 ^bcd^	21.37^d−g^	7.70 ^a−e^	6.73 ^a^	11.00 ^bcd^	12.00 ^a^	42.67 ^a−d^	39.33 ^a^	4.17 ^gh^	3.37 ^cde^
	VI	21.73 ^f−j^	21.4 ^d−g^	7.07 ^b−g^	7.60 ^a^	9.00 ^g^	9.33 ^de^	34.67 ^a−d^	31.67 ^ab^	3.57 ^i^	3.1 ^fg^
	LI	22.73 ^e−h^	21.60 ^d−g^	8.27 ^abc^	6.93 ^a^	7.67 ^h^	9.33 ^de^	37.33 ^a−d^	34 ^ab^	3.47 ^ij^	3.17 ^efg^
	VLI	22.57 ^f−i^	22.97 ^b−f^	6.47 ^efg^	6.63 ^a^	10.00 ^d−g^	11.33^ab^	40.67 ^a−d^	38.67 ^a^	4.57 ^bcd^	3.33 ^cde^
S3	C	16.77 ^jk^	12.60 ^j^	6.43^efg^	5.50 ^a^	9.00 ^g^	9.33 ^de^	26.00 ^d^	28 ^ab^	2.43 ^m^	2.23 ^k^
	V	18.33^g−k^	17.33^ghi^	6.57^efg^	5.83 ^a^	10.67 ^b−e^	11.00 ^abc^	30.00 ^bcd^	22.67 ^b^	2.70 ^kl^	2.33 ^jk^
	L	17.2 ^jk^	14.00 ^ij^	6.27 ^fg^	6.80 ^a^	12.33 ^a^	10.33 ^bcd^	40.00 ^a−d^	31 ^ab^	2.90 ^k^	2.97 ^gh^
	I	16.53 ^k^	19.13 ^fgh^	6.03 ^g^	6.43 ^a^	9.67 ^efg^	10.00 ^cd^	29.33 ^cd^	31.33^ab^	2.53 ^lm^	2.83 ^h^
	VL	15.67 ^k^	22.83 ^b−f^	6.27 ^fg^	6.03 ^a^	10.67 ^b−e^	11.33 ^ab^	34.33 ^a−d^	32 ^ab^	2.43 ^m^	2.60 ^i^
	VI	17.63 ^ijk^	19.33 ^fgh^	7.03 ^c−g^	7.07 ^a^	9.67 ^efg^	8.00 ^f^	30.67 ^bcd^	28 ^ab^	2.60 ^lm^	2.87 ^h^
	LI	18.70 ^g−k^	15.53 ^hij^	6.70 ^d−g^	6.50 ^a^	10.00 ^d−g^	8.00 ^f^	31.00 ^bcd^	28.33^ab^	2.57 ^lm^	2.40 ^ijk^
	VLI	19.07^g−k^	22.03 ^c−f^	6.03 ^g^	6.37 ^a^	9.33 ^fg^	10.33 ^bcd^	32.00 ^a−d^	33.33^ab^	3.3 ^j^	3.43 ^bcd^
HSD A*B at the 5%		9.90	10.06	2.56	2.55	1.15	1.14	21.48	15.48	0.21	0.23

Means followed by the same letter within the same column do not differ significantly according to the Tukey HSD test at the 5% significance level. Where: S0 = 0 ppm NaCl [Control]; S1.5 = 1500 ppm NaCl; S3 = 3000 ppm NaCl; C= Control (spray water); V= Valine (2 g L⁻¹); L= Leucine (2 g L⁻¹); I= Isoleucine (2 g L⁻¹); VL= Valine (1 g L⁻¹)+ Leucine (1 g L⁻¹); VI= Valine (1 g L⁻¹)+Isoleucine (1 g L⁻¹); LI= Leucine (1 g L⁻¹)+ Isoleucine (1 g L⁻¹); VLI= Valine (0.75 g L⁻¹)+ Leucine (0.75 g L⁻¹)+ Isoleucine (0.75 g L⁻¹).

Stem diameter was influenced by both salinity and BCAA treatments in both seasons (Table 5). In general, stem diameter declined with increasing salinity, with the lowest values consistently recorded under severe salinity (S3). Under non-saline conditions (S0), foliar application of BCAAs, particularly valine, leucine, and their combinations, improved stem diameter compared with the untreated control. Under moderate salinity (S1.5), treatment responses were variable, while severe salinity markedly suppressed stem development regardless of amino acid application.

The number of stems per plant also varied in response to salinity and BCAA treatments (Table 5). Moderate salinity combined with certain amino acid treatments promoted stem production in both seasons, whereas severe salinity generally reduced stem number. Although some BCAA treatments partially alleviated the negative effects of salinity, treatment responses varied between seasons.

Similarly, total tuber number per plant was strongly affected by salinity and foliar amino acid application (Table 5). Moderate salinity combined with isoleucine or amino acid combinations produced the highest tuber numbers in both seasons, whereas severe salinity substantially reduced tuber formation. The lowest tuber numbers were consistently recorded under S3, particularly in untreated plants, indicating that high salinity strongly inhibited tuber initiation despite the mitigating effects of BCAA treatments.

Data in Table (5) indicate that total tuber weight was significantly influenced by salinity level and BCAA foliar application across both seasons. Overall, a clear declining trend in tuber weight was observed with increasing salinity, with S0 consistently producing the highest values and S3 the lowest, regardless of treatment. Under non-saline conditions (S0), BCAA treatments, particularly V, L, and VLI, maintained comparatively higher tuber weights, with only minor and non-significant differences among the best-performing treatments. In contrast, salinity stress reduced tuber weight across all treatments, with the greatest reductions recorded under S3, where control plants consistently showed the lowest performance.

In the 2023 season, the highest tuber weights were observed under S0 for V and VLI, while intermediate responses were recorded under S0 and S1.5 with overlapping statistical groupings, indicating limited separation among several treatments under mild to moderate stress. The most pronounced reductions occurred under S3, where C and VL showed the lowest values. A similar pattern was observed in 2024, with L and VL under S0 showing the highest values, while S3 again resulted in minimum tuber weights, particularly in the control treatment. Across both seasons, L- and combination-based treatments tended to sustain relatively higher tuber weights under stress conditions, suggesting partial mitigation of salinity-induced yield reduction.

### Yield components and tuber grading

Data presented in Table (6) showed that the number and weight of small tubers (≤ 25 mm) were significantly affected by salinity level and BCAA foliar application across both seasons. Overall, increasing salinity consistently reduced the formation and biomass of small tubers, with S0 and S1.5 generally maintaining higher values compared with S3. Differences among BCAA treatments were more pronounced under non- or moderately saline conditions, whereas severe salinity (S3) markedly suppressed all responses.

For small tuber number, relatively higher values were generally recorded under S0 and S1.5, with V, I, and VLI often among the top-performing treatments depending on the season. In contrast, S3 consistently produced the lowest numbers, particularly in the control (C) and LI treatments in 2023, and again in C in 2024, indicating a strong inhibitory effect of high salinity. A similar pattern was observed for small tuber weight, where I and VLI under S1.5 frequently showed relatively higher values, while S3 resulted in uniformly low weights across treatments, with minimal variation among them.

Table (6) also indicated that the number of medium-sized tubers (25–55 mm) was strongly influenced by both salinity and BCAA application. Higher production was generally associated with S0 and S1.5, where L, V, VI, and I frequently recorded comparable top values with no consistent significant separation among the best-performing treatments. In contrast, S3 consistently reduced medium tuber number across all treatments, with VLI and C often among the lowest. This trend was consistent in both seasons, confirming a salinity-driven reduction in marketable tuber formation.

A similar response was observed for medium tuber weight, where higher values were generally maintained under S0, particularly with VI, V, L, and VLI depending on the season. Severe salinity (S3) significantly reduced tuber weight, with LI and VI among the lowest recorded treatments, indicating limited effectiveness of BCAA application under high stress conditions.

Regarding large tubers (≥ 55 mm), results showed a clear suppression effect of salinity. S0 consistently supported higher numbers, with V, VL, L, and I frequently showing the greatest values, although differences among top treatments were generally minor. In contrast, S3 led to a drastic reduction or complete absence of large tubers in several treatments, particularly VL, VI, and VLI in 2024, indicating that severe salinity can completely inhibit large tuber formation regardless of BCAA application.

**Table 6:** Effect of foliar application of branched-chain amino acids on yield parameters of potato plants under saline irrigation conditions during the 2023 and 2024 growing seasons.

Treatments		No. of tuber ≤ 25 per plant		Weight of tuber ≤ 25 per plant (g.)		No. of tuber 25:55 per plant		Weight of tuber 25:55 per plant (g.)		No. of tuber ≥ 55 per plant		weight of tuber ≥ 55 per plant (g.)	
		2023	2024	2023	2024	2023	2024	2023	2024	2023	2024	2023	2024
S0	C	15.00 ^abc^	8.67 ^ab^	0.56 ^a−d^	0.40 ^ab^	18.33 ^a^	19.33^a^	2.16 ^d−g^	2.36 ^ab^	3.67 ^a−d^	2.00 ^a^	0.89 ^abc^	0.51 ^b^
	V	23.33 ^ab^	8.67 ^ab^	0.53 ^a−d^	0.37 ^ab^	23.33 ^a^	17.67 ^a^	3.29^abc^	2.47 ^ab^	4.67 ^a^	3.33 ^a^	1.07 ^ab^	0.79 ^b^
	L	15.33^abc^	9.67 ^ab^	0.38 ^a−d^	0.44 ^ab^	26.67 ^a^	23.00 ^a^	3.27 ^abc^	2.75 ^ab^	4 ^abc^	4.00 ^a^	1.01 ^abc^	0.91 ^b^
	I	19.33^abc^	8.67 ^ab^	0.57 ^a−d^	0.37 ^ab^	25.33 ^a^	20.33 ^a^	3.08 ^a−e^	2.66 ^ab^	4.33 ^ab^	1.67 ^a^	1.10 ^ab^	0.60 ^b^
	VL	14.33^abc^	11.00^ab^	0.47 ^a−d^	0.46 ^ab^	22.33 ^a^	20.00 ^a^	2.67 ^a−g^	2.72 ^ab^	4.33 ^ab^	2.67 ^a^	1.17 ^a^	0.84 ^b^
	VI	10.67 ^bc^	7.67 ^b^	0.51 ^a−d^	0.46 ^ab^	26.33 ^a^	19.00 ^a^	3.44 ^a^	2.67 ^ab^	1.33 ^a−d^	3.00 ^a^	0.45 ^abc^	0.89 ^b^
	LI	8.00 ^c^	7.33 ^b^	0.44 ^a−d^	0.37 ^ab^	22.33 ^a^	18.00 ^a^	3.12 ^a−d^	2.60 ^ab^	3 ^a−d^	1.67 ^a^	0.94 ^abc^	0.53 ^b^
	VLI	11.67 ^abc^	9.67 ab	0.60 ^a−d^	0.53 ^ab^	19.00 ^a^	17.67 ^a^	3.27 ^abc^	3.36 ^a^	2.67 ^a−d^	0.00 ^a^	1.02 ^abc^	0.00 ^b^
S1.5	C	11.67 ^abc^	8.00 ^ab^	0.29 ^cd^	0.27 ^ab^	20.00 ^a^	20.00 ^a^	2.45^a−g^	2.50 ^ab^	0.33 ^cd^	0.33 ^a^	0.11 ^bc^	4.17 ^ab^
	V	12.67 ^abc^	10.00 ^ab^	0.38 ^a−d^	0.34 ^ab^	24.33 ^a^	27.00 ^a^	2.7 ^a−g^	2.75 ^ab^	1.33 ^a−d^	0.67 ^a^	0.54 ^abc^	5.01 ^ab^
	L	14.67 ^abc^	9.67 ^ab^	0.47 ^a−d^	0.22 ^b^	25.67 ^a^	23.00 ^a^	2.76^a−g^	2.81 ^ab^	2 ^a−d^	1.33 ^a^	0.75 ^abc^	10.36 ^ab^
	I	25.33 ^a^	10.67 ^ab^	0.82 ^a^	0.30 ^ab^	26.33 ^a^	20.67 ^a^	2.90 ^a−f^	2.95 ^ab^	1.33 ^a−d^	2.00 ^a^	0.48 ^abc^	15.10 ^a^
	VL	16.33 ^abc^	13.67 ^ab^	0.67 ^abc^	0.45 ^ab^	23.67 ^a^	25.67 ^a^	2.70 ^a−g^	2.75 ^ab^	2.67 ^a−d^	0.00 ^a^	0.78 ^abc^	0.00 ^b^
	VI	13.00 ^abc^	9.00 ^ab^	0.55 ^a−d^	0.23 ^b^	19.67 ^a^	22.67 ^a^	2.37^b−g^	2.42 ^ab^	2 ^a−d^	0.00 ^a^	0.65 ^abc^	0.00 ^b^
	LI	12.00 ^abc^	13.00 ^ab^	0.45 ^a−d^	0.48 ^ab^	23.67 ^a^	20.33 ^a^	2.47^a−g^	2.52 ^ab^	1.67 ^a−d^	0.67 ^a^	0.53 ^abc^	5.69 ^ab^
	VLI	15.00 ^abc^	18.67 ^a^	0.77 ^ab^	0.65 ^a^	24.33 ^a^	20.00 ^a^	3.41 ^ab^	3.46 ^a^	1.33 ^a−d^	0.00 ^a^	0.40 ^abc^	0.00 ^b^
S3	C	7.00 ^c^	6.33 ^b^	0.20 ^d^	0.20 ^b^	18.33 ^a^	21.67 ^a^	2.07 ^d−g^	2.05 ^ab^	0.67 ^bcd^	0.00 ^a^	0.17^abc^	0.00 ^b^
	V	8.67 ^c^	7.67 ^b^	0.22 ^cd^	0.29 ^ab^	21.00 ^a^	14.00 ^a^	2.35 ^b−g^	1.77 ^b^	0.33 ^cd^	1.00 ^a^	0.11 ^bc^	0.27 ^b^
	L	15.33 ^abc^	10.33 ^ab^	0.43 ^a−d^	0.32 ^ab^	24.00 ^a^	18.67 ^a^	2.32 ^c−g^	2.20 ^ab^	0.67 ^bcd^	2.00 ^a^	0.15 ^abc^	0.45 ^b^
	I	10.33 ^bc^	12.67 ^ab^	0.33 ^bcd^	0.45 ^ab^	18.00 ^a^	17.67 ^a^	1.87 ^fg^	2.15 ^ab^	1.00 ^a−d^	1.00 ^a^	0.30 ^abc^	0.22 ^b^
	VL	14.33 ^abc^	9.67 ^ab^	0.4 ^a−d^	0.32 ^ab^	20.00 ^a^	22.33 ^a^	2.02 ^efg^	2.27 ^ab^	0.00 ^d^	0.00 ^a^	0.00 ^c^	0.00 ^b^
	VI	14.33 ^abc^	8.33 ^ab^	0.43 ^a−d^	0.35 ^ab^	20.67 ^a^	18.00 ^a^	1.82 ^g^	2.05 ^ab^	1.33^a−d^	1.67 ^a^	0.35 ^abc^	0.45 ^b^
	LI	9.33 ^bc^	11.00 ^ab^	0.33 ^bcd^	0.37 ^ab^	21.33 ^a^	16.00 ^a^	2.00 ^fg^	1.73 ^b^	1.00 ^a−d^	1.33 ^a^	0.23 ^abc^	0.32 ^b^
	VLI	10.33 ^bc^	13.67 ^ab^	0.51 ^a−d^	0.47 ^ab^	15.00 ^a^	19.33 ^a^	2.67 ^a−g^	2.84 ^ab^	0.33 ^cd^	0.33 ^a^	0.13 ^bc^	0.11 ^b^
HSD A*B at the 5%		14.36	10.78	0.45	0.40	13.76	16.04	1.07	1.42	3.85	4.53	1.03	12.95

Means followed by the same letter within the same column do not differ significantly according to the Tukey HSD test at the 5% significance level. Where: S0 = 0 ppm NaCl [Control]; S1.5 = 1500 ppm NaCl; S3 = 3000 ppm NaCl; C= Control (spray water); V= Valine (2 g L⁻¹); L= Leucine (2 g L⁻¹); I= Isoleucine (2 g L⁻¹); VL= Valine (1 g L⁻¹)+ Leucine (1 g L⁻¹); VI= Valine (1 g L⁻¹)+Isoleucine (1 g L⁻¹); LI= Leucine (1 g L⁻¹)+ Isoleucine (1 g L⁻¹); VLI= Valine (0.75 g L⁻¹)+ Leucine (0.75 g L⁻¹)+ Isoleucine (0.75 g L⁻¹).

Regarding the weight of large tubers (≥ 55 mm), results in Table (6) showed a clear stimulatory effect of BCAA application under non- and moderately saline conditions, while severe salinity markedly suppressed large tuber formation. In general, S1.5 supported the highest large tuber weights across treatments, whereas S3 consistently resulted in minimal or zero values.

In the 2023 season, Isoleucine (I) under S1.5 produced the highest large tuber weight (15.10 g), while V and LI under S1.5 also recorded relatively elevated values (5.01 and 5.69 g, respectively), with no consistent significant separation among several treatments within this salinity level. In contrast, S3 resulted in complete elimination of large tuber weight in C, VL, and VI treatments, indicating strong inhibition under severe stress. A similar pattern was observed in 2024, where I under S1.5 again recorded the highest value (15.10 g), clearly outperforming all other treatments across both seasons, while VL, VI, and VLI under S1.5 and S3 produced zero values, confirming the strong suppressive effect of high salinity on large tuber development.

**Fig. 2:**
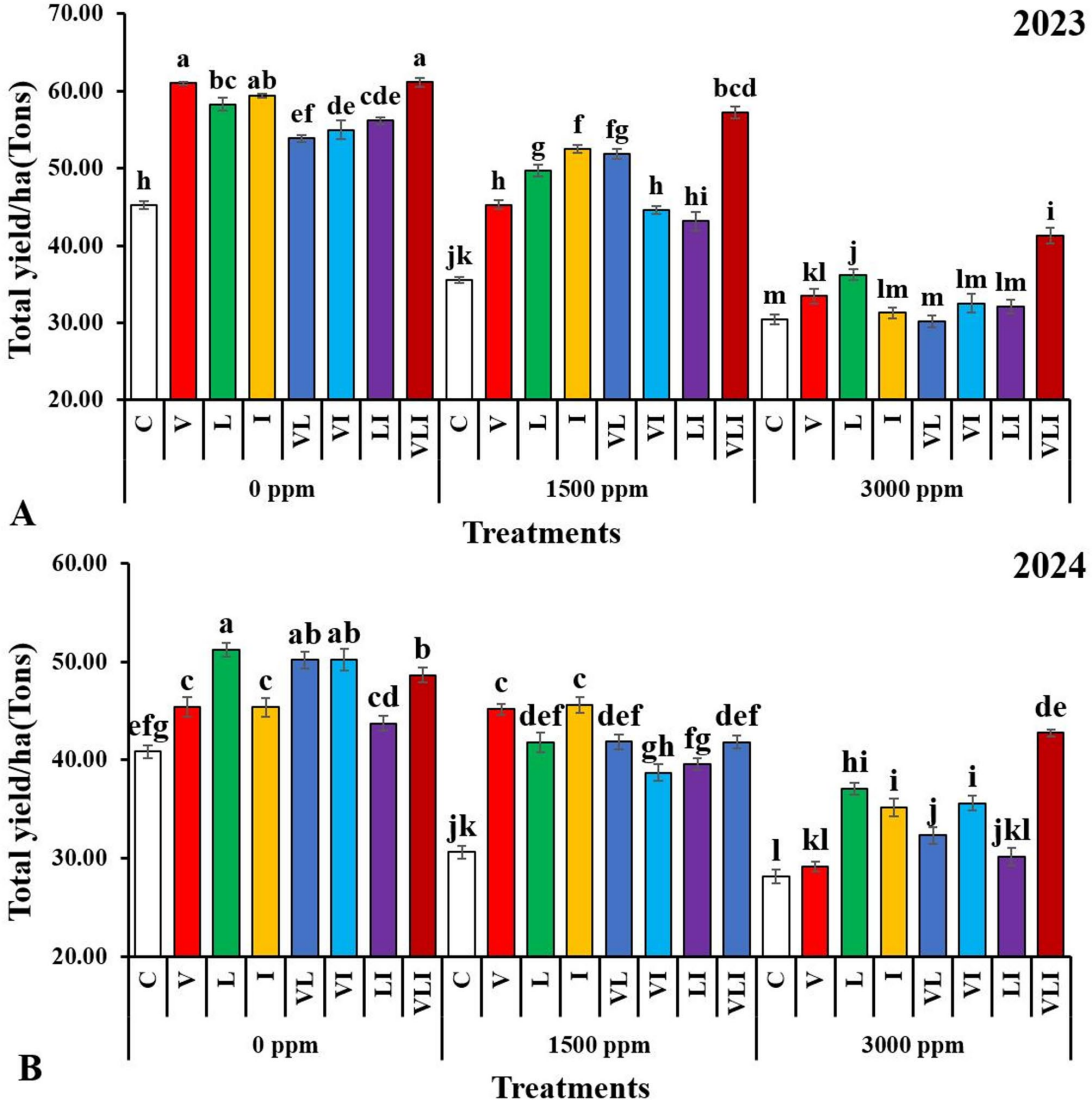
Effect of foliar application of branched-chain amino acids on total yield ha^− 1^ (Tons) of potato plants under saline irrigation conditions during the 2023 (**A**) and 2024 (**B**) growing seasons. Values are means ± SD. Bars topped with the same letter within each column do not differ significantly according to the Tukey HSD test at the 5% significance level. Where: S0 = 0 ppm NaCl [Control]; S1.5 = 1500 ppm NaCl; S3 = 3000 ppm NaCl; C= Control (spray water); V= Valine (2 g L⁻¹); L= Leucine (2 g L⁻¹); I= Isoleucine (2 g L⁻¹); VL= Valine (1 g L⁻¹)+ Leucine (1 g L⁻¹); VI= Valine (1 g L⁻¹)+Isoleucine (1 g L⁻¹); LI= Leucine (1 g L⁻¹)+ Isoleucine (1 g L⁻¹); VLI= Valine (0.75 g L⁻¹)+ Leucine (0.75 g L⁻¹)+ Isoleucine (0.75 g L⁻¹).

The total potato yield per feddan (Fig. 2) was significantly affected by the interaction between salinity level and BCAA foliar application, showing a consistent yield decline with increasing salinity, alongside a partial mitigation effect of amino acid treatments. Under non-saline conditions, yield ranged from 41.25 tons ha⁻¹ in the control to higher values under BCAA treatments, with V (51.57 tons ha⁻¹) and I (49.98 tons ha⁻¹) among the highest performers, while most combinations (VL, VI, VLI) also maintained comparatively elevated yields with limited separation among them. Under moderate salinity (1500 ppm NaCl), the control yield decreased to 29.35 tons ha⁻¹, whereas BCAA application substantially alleviated this reduction, particularly with V and I, which maintained relatively high yields, and VLI, which also showed strong performance compared with the untreated control. Under severe salinity (3000 ppm NaCl), yield further declined in the control (27.77 tons ha⁻¹), but BCAA treatments still improved productivity relative to the control. L showed a comparatively higher value (36.49 tons ha⁻¹), followed by VI and VLI, indicating partial stress mitigation even under high salinity. Overall, VLI showed the most stable performance across salinity levels, reflecting the strongest capacity to maintain yield under stress conditions, while single amino acids—particularly V and I—were more effective under non- and moderate salinity than under severe stress.

### Biochemical responses

Data presented in Table (7) showed that proline content in potato leaves was significantly influenced by salinity level and BCAA foliar application across both seasons, with a consistent overall pattern of increasing proline accumulation under higher salinity stress. In general, S3 induced the greatest proline accumulation, while S0 maintained the lowest levels, regardless of treatment. In the 2023 season, relatively higher proline values under non-saline conditions were observed in VI and LI under S0, with no significant difference between them, while moderate salinity (S1.5) resulted in an overall increase in proline, particularly in LI and VLI. In contrast, the lowest values were recorded under S0 in C and V, reflecting minimal stress conditions and limited osmotic adjustment requirements. A clearer salinity-driven response was observed in 2024, where severe salinity (S3) led to a marked increase in proline accumulation, with VLI and LI recording the highest values, followed by VI. Conversely, the lowest proline levels were consistently recorded under S0, particularly in I and C, confirming that proline accumulation was strongly and positively associated with salinity intensity.

**Table 7:** Effect of foliar application of branched-chain amino acids on biochemical parameters of potato plants under saline irrigation conditions during the 2023 and 2024 growing seasons.

Treatments		Proline content µg g⁻¹		Carbohydrate content mg g⁻¹ DW		Protein content mg g⁻¹ DW		Catalase content µmol min⁻¹mg⁻¹ protein		Peroxidase content µmol min⁻¹g⁻¹ FW	
		2023	2024	2023	2024	2023	2024	2023	2024	2023	2024
S0	C	0.59 ^hi^	0.53 ^kl^	0.61 ^ij^	0.56 ^h^	0.15 ^m^	0.17 ^l^	0.16 ^l^	0.15 ^l^	0.22^jk^	0.21 ^jk^
	V	0.27 ^l^	0.5 ^l^	0.98 ^a^	0.95 ^a^	0.29 ^j−m^	0.31 ^kl^	0.29 ^ij^	0.39 ^h^	0.49 ^hi^	0.47 ^ghi^
	L	0.58 ^hi^j	0.57 ^i−l^	0.89 ^b^	0.59 ^g^	0.18 ^lm^	0.20 ^l^	0.29 ^ij^	0.29 ^i^	0.28 ^j^	0.27 ^j^
	I	0.51 ^ijk^	0.26 ^m^	0.82 ^c^	0.78 ^c^	0.35 ^i−l^	0.37 ^kl^	0.32 ^hi^	0.31 ^i^	0.47 ^hi^	0.45 ^ghi^
	VL	0.45 ^jk^	0.43 ^l^	0.95 ^a^	0.67 ^e^	0.39 ^ij^	0.41 ^i−l^	0.21 ^kl^	0.20 ^kl^	0.43 ^i^	0.4 ^i^
	VI	1.48 ^a^	0.72 ^ghi^	0.71 ^e^	0.68 ^de^	0.74 ^fg^	0.76 ^fgh^	0.27 ^ijk^	0.26 ^ij^	0.51 ^gh^	0.5 ^gh^
	LI	1.47 ^a^	1.26 ^c^	0.7 ^ef^	0.68 ^e^	0.48 ^hi^	0.48 ^g−l^	0.28 ^ijk^	0.27 ^ij^	0.52 ^gh^	0.51 ^g^
	VLI	1.14 ^cd^	1.09 ^de^	0.71 ^e^	0.68 ^e^	0.37 ^ijk^	0.33 ^jkl^	0.27 ^ijk^	0.26 ^ijk^	0.48 ^hi^	0.46 ^ghi^
S1.5	C	0.27 ^l^	0.56 ^i−l^	0.59 ^j^	0.50 ^j^	0.20 ^klm^	0.34 ^l^	0.17 ^l^	0.16 ^l^	0.2 ^k^	0.19 ^k^
	V	1.16 ^bcd^	0.74 ^gh^	0.77 ^d^	0.86 ^b^	0.59 ^gh^	0.61 ^g−k^	0.29 ^ij^	0.58 ^bc^	0.73 ^de^	0.75 ^cde^
	L	0.59 ^hi^	0.56 ^jkl^	0.71 ^e^	0.53 ^i^	0.35 ^ijk^	0.37 ^kl^	0.5 ^de^	0.43 ^gh^	0.42 ^i^	0.44 ^hi^
	I	0.74 ^fg^	0.71 ^g−j^	0.71 ^e^	0.70 ^d^	0.70 ^fg^	0.72 ^f−j^	0.55 ^d^	0.46 ^fg^	0.71 ^ef^	0.72 ^e^
	VL	0.69 ^gh^	0.67 ^h−k^	0.81 ^c^	0.6 ^fg^	0.78 ^f^	0.80 ^fg^	0.36 ^gh^	0.30 ^i^	0.64 ^f^	0.65 ^f^
	VI	0.89 ^e^	0.86 ^fg^	0.69 ^ef^	0.62 ^f^	1.47 ^c^	1.49 ^cd^	0.47 ^e^	0.40 ^h^	0.77 ^de^	0.79 ^bcd^
	LI	1.21 ^bc^	1.42 ^b^	0.63 ^hij^	0.61 ^f^	0.97 ^e^	0.99 ^ef^	0.50 ^de^	0.40 ^h^	0.78 ^cd^	0.81 ^bc^
	VLI	1.27 ^b^	1.21 ^cd^	0.69 ^efg^	0.61 ^f^	0.73 ^fg^	0.64 ^fgh^	0.46 ^ef^	0.38 ^h^	0.71 ^de^	0.73 ^de^
S3	C	0.39 ^kl^	1.14 ^cde^	0.53 ^k^	0.38 ^m^	0.40 ^ij^	0.65 ^h−l^	0.24 ^jk^	0.23 ^jk^	0.23 ^jk^	0.28 ^j^
	V	0.64 ^ghi^	1.09 ^de^	0.59 ^j^	0.67 ^e^	1.18 ^d^	1.09 ^de^	0.40 ^fg^	0.78 ^a^	0.98 ^ab^	0.85 ^ab^
	L	1.21 ^bc^	1.17 ^cde^	0.6 ^ij^	0.41 ^l^	0.71 ^fg^	0.73 ^f−i^	0.63 ^c^	0.57 ^bcd^	0.56 ^g^	0.49 ^gh^
	I	1.05 ^d^	1.02 ^ef^	0.66 ^fgh^	0.55 ^hi^	1.41 ^c^	1.43 ^cd^	0.41 ^fg^	0.62 ^b^	0.94 ^b^	0.81 ^bc^
	VL	0.87 ^ef^	0.85 ^g^	0.70 ^ef^	0.47 ^k^	1.55 ^c^	1.57 ^c^	0.61 ^c^	0.41 ^h^	0.85 ^c^	0.73 ^de^
	VI	0.77 ^efg^	1.43 ^b^	0.51 ^k^	0.48 ^k^	2.95 ^a^	2.97 ^a^	0.95 ^ab^	0.53 ^de^	1.03 ^a^	0.89 ^a^
	LI	1.19 ^bc^	1.75 ^a^	0.59 ^j^	0.48 ^k^	1.93 ^b^	1.95 ^b^	1.01 ^a^	0.53 ^cde^	1.05 ^a^	0.91 ^a^
	VLI	1.45 ^a^	1.81 ^a^	0.64 ^ghi^	0.47 ^k^	1.47 ^c^	1.49 ^cd^	0.92 ^b^	0.51 ^ef^	0.95 ^b^	0.82 ^bc^
HSD A*B at the 5%		0.13	0.16	0.05	0.022	0.18	0.34	0.06	0.05	0.07	0.07

Means followed by the same letter within the same column do not differ significantly according to the Tukey HSD test at the 5% significance level. Where: S0 = 0 ppm NaCl [Control]; S1.5 = 1500 ppm NaCl; S3 = 3000 ppm NaCl; C= Control (spray water); V= Valine (2 g L⁻¹); L= Leucine (2 g L⁻¹); I= Isoleucine (2 g L⁻¹); VL= Valine (1 g L⁻¹)+ Leucine (1 g L⁻¹); VI= Valine (1 g L⁻¹)+Isoleucine (1 g L⁻¹); LI= Leucine (1 g L⁻¹)+ Isoleucine (1 g L⁻¹); VLI= Valine (0.75 g L⁻¹)+ Leucine (0.75 g L⁻¹)+ Isoleucine (0.75 g L⁻¹).

Data presented in Table (7) showed that proline content in potato leaves was significantly affected by salinity level and BCAA foliar application across both growing seasons, with a consistent overall trend of increasing accumulation under higher salinity stress. In general, proline levels were lowest under S0, increased under S1.5, and reached their highest values under S3, indicating a clear stress-induced osmoprotective response. In the 2023 season, relatively higher values under S0 were observed in VI and LI with no significant difference between them, while moderate salinity (S1.5) led to an increase in several treatments, particularly LI and VLI. In contrast, the lowest proline values were consistently recorded under S0 in C and V, reflecting minimal stress conditions and limited need for osmotic adjustment. A more pronounced response was observed in 2024, where severe salinity (S3) resulted in the highest proline accumulation, particularly in VLI and LI, followed by VI, confirming enhanced osmotic regulation under stress. Conversely, the lowest values were recorded under S0, especially in I and C, further supporting the strong positive relationship between salinity intensity and proline accumulation.

Data presented in Table (7) indicated that carbohydrate content in potato leaves was significantly influenced by salinity level and BCAA foliar application, with a general decline in carbohydrate accumulation as salinity increased. Overall, S0 consistently maintained the highest carbohydrate levels, whereas S3 recorded the lowest values across treatments in both seasons. In the 2023 season, the highest carbohydrate content was observed under S0, particularly in V, VL, and L, with no significant differences among several S0 treatments. A gradual reduction was evident under S1.5 and became more pronounced under S3, where the control recorded the minimum value. A similar pattern was observed in 2024, with V and VL under S0 showing the highest carbohydrate contents and no clear separation between them. Across both seasons, severe salinity (S3) consistently reduced carbohydrate accumulation, with the lowest values recorded in the control and VI treatments. Despite this reduction, BCAA application—particularly Valine and its combination with Leucine (VL)—helped sustain comparatively higher carbohydrate levels under stress conditions, indicating a partial mitigation of salinity-induced inhibition of carbohydrate synthesis.

As shown in Table (7), protein content in potato leaves was significantly affected by the interaction between salinity level and BCAA foliar application across both growing seasons. Overall, protein accumulation increased under salinity stress, particularly under S3, indicating activation of stress-related metabolic responses. In 2023, higher protein contents were generally associated with VI, LI, and VL treatments under moderate and severe salinity, whereas the control consistently recorded the lowest values regardless of salinity level. In 2024, the stimulatory effect of salinity on protein accumulation became more pronounced, with S3 producing markedly higher protein levels than S0 and S1.5. Among the BCAA treatments, VI and LI consistently maintained the highest protein contents under severe salinity, while untreated plants showed comparatively limited protein accumulation.

Data in Table (7) showed that catalase activity was significantly influenced by salinity level and BCAA foliar application across both seasons, with a general increase in enzymatic activity under salinity stress, particularly at higher levels. Overall, S3 tended to induce greater catalase activity compared with S0, indicating enhanced antioxidant defense under severe stress conditions. In the 2023 season, the highest catalase activity was recorded under S3, particularly in LI and VI treatments, while VLI under S0 showed intermediate activity. In contrast, the lowest values were observed in the control under S3 and VL under S0, reflecting reduced antioxidant response in the absence of effective BCAA stimulation. Intermediate responses were widely distributed across S0 and S1.5, with considerable overlap among treatments. A similar pattern was observed in 2024, where S3 again induced the highest catalase activity, with V, LI, and VI showing comparatively elevated and statistically comparable values. The lowest catalase activity was recorded in the control under S0 and S3, indicating weaker antioxidant defense in untreated plants.

Results in Table (7) showed that peroxidase activity was significantly affected by salinity level and BCAA foliar application across both seasons, with a general increase in activity under salinity stress, particularly at higher levels. Overall, S3 tended to induce greater peroxidase activity compared with S0, indicating activation of antioxidant defense responses under severe stress conditions. In the 2023 season, the highest peroxidase activity was recorded under S3, mainly in LI and VI, while LI under S0 showed comparatively moderate values. In contrast, the lowest activity was observed under S0, particularly in the control and VL treatments, reflecting weaker antioxidant defense under non-stressed conditions. Intermediate values were distributed across S0 and S1.5 with noticeable overlap among treatments. A similar trend was observed in 2024, where S3 again supported relatively high peroxidase activity, with VI and V showing comparable values, while LI under S1.5 also recorded a relatively elevated response. The lowest peroxidase activity was consistently recorded in the control under both S0 and S3, confirming reduced enzymatic defense in untreated plants.

### Pearson correlation and principal component analysis

**Fig. 3:**
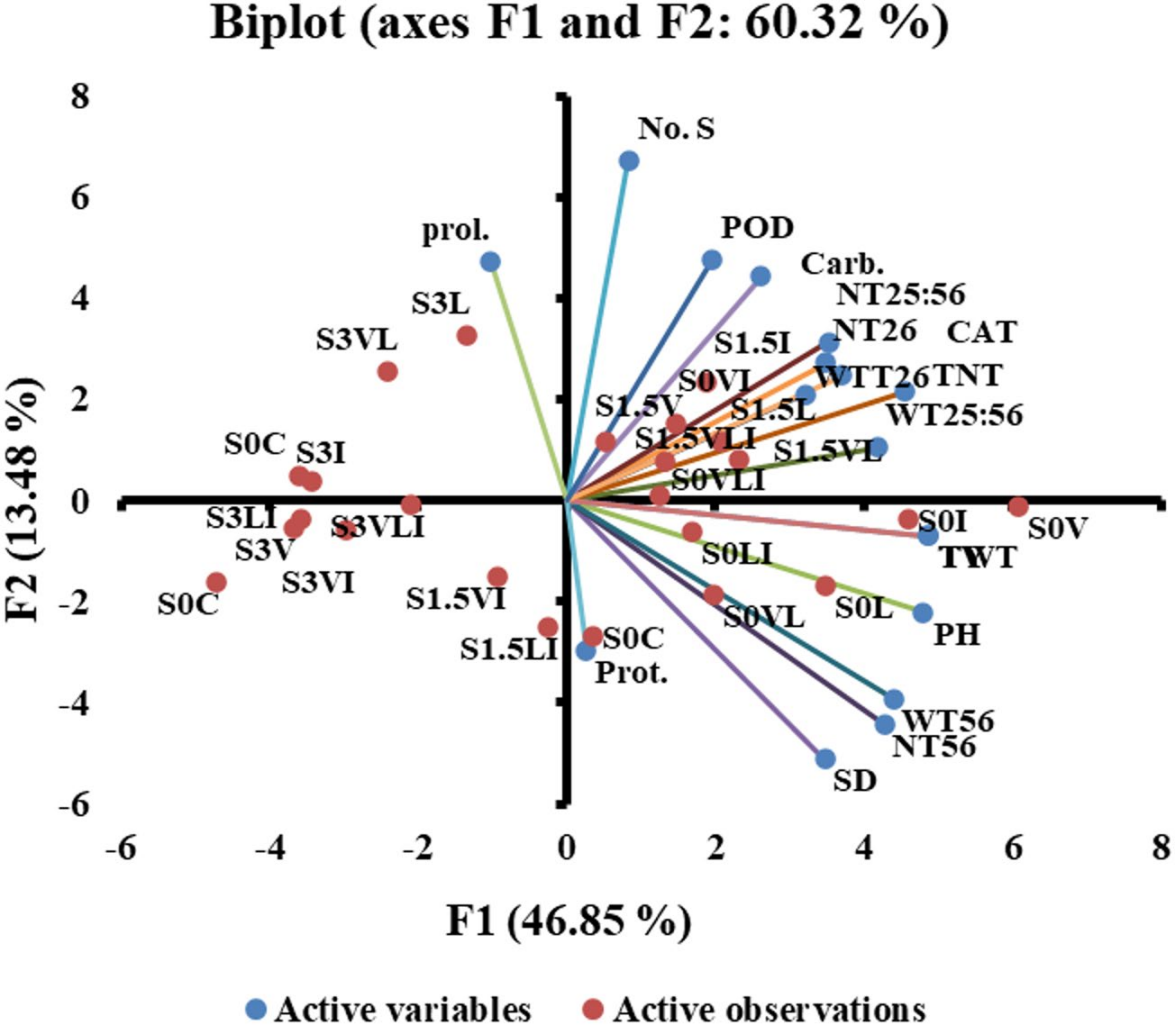
Biplot of principal component analysis (PCA) for growth, yield, and biochemical traits during the first growing season, illustrating trait associations and treatment clustering. Treatment abbreviations are shown in Table 2. Where: PH=Plant height; SD= Stem diameters; No. S = No. of Stem; NT26 = No. of tuber ≤ 25; WTT26 = Weight of tuber ≤ 25 per plant; NT25:56 = No. of tuber 25:55; WT25:56 = Weight of tuber 25:55 per plant; NT56 = No. of tuber ≥ 55 per plant; WT56 = Weight of tuber ≥ 55 per plant; TNT= Total No. of tubers; TWT= Total weight of tubers; TY= tTotal yield feddan^− 1^; prol.= Proline content; Carb.= Carbohydrate content; Prot.= Protein content; CAT= Catalase content; POD= Peroxidase content.

Principal component analysis with Varimax rotation was performed using XLSTAT to explore the integrated relationships among vegetative growth, yield components, and biochemical traits of potato plants under different salinity levels and BCAA foliar treatments (Fig. 3). In both growing seasons (2023 and 2024), two components with eigenvalues greater than 1.0 were extracted, explaining 60.33% of the total variance (PC1: 46.85%, PC2: 13.48%). After Varimax rotation, PC1 was primarily loaded by plant height (PH), total yield (TY), total tuber weight (TWT), total tuber number (TNT), and weight of large tubers (WT56), representing a growth-yield dimension. PC2 was loaded by proline content, catalase (CAT), and peroxidase (POD), representing a stress-tolerance dimension.

The PCA biplot clearly separated treatments under different salinity levels. Under severe salinity (S3), combined BCAA treatments — particularly VLI — clustered toward higher PC2 scores (elevated proline, CAT, and POD), indicating enhanced antioxidant defense and osmotic adjustment. In contrast, untreated controls under S3 clustered toward lower PC2 scores, reflecting weaker stress tolerance. Under non-saline (S0) and moderate salinity (S1.5), treatments with individual amino acids (V, L, I) and dual combinations showed intermediate clustering patterns, consistent with their moderate protective effects. These results confirm that BCAA-mediated salinity tolerance operates through coordinated enhancement of both agronomic performance and physiological defense mechanisms, with synergistic combinations (VLI) providing the greatest protection under severe salt stress.

## Discussion

The observed decline in vegetative growth, yield performance, and tuber quality under saline conditions may be attributed to the combined effects of osmotic stress, ion toxicity, and nutrient imbalance, which restrict water uptake, cell expansion, and overall plant development^34^. Increased NaCl levels can disrupt cell division and elongation, impair metabolic activity, and reduce photosynthetic efficiency, ultimately limiting plant growth and productivity. In addition, excessive accumulation of Na⁺ and Cl⁻ ions may interfere with the uptake of essential nutrients, further aggravating growth inhibition under saline conditions^5,35^. These adverse effects of salinity on potato growth and physiological performance are consistent with earlier reports by Balasubramaniam, et al.^12^ in various crop species, Masarmi, et al.^36^ in wheat crop, and by Seymen, et al.^37^ in cabbage seedlings under arid environments.

The decrease in tuber number and total yield under salinity—particularly at 3000 ppm—reflects the sensitivity of potato to salt stress during both vegetative and tuberization stages. Similar yield reductions under salinity have been documented by Chourasia, et al. ^9^ and Sanwal, et al.^35^, who reported that potato cultivars exhibit substantial yield losses when electrical conductivity exceeds 2.5 dS m⁻¹. In the present study, salinity likely limited assimilate production and translocation, resulting in reduced tuber initiation and bulking, which aligns with findings by George, et al.^38^ and Al-Taey and Hussain^39^ under drought and salt stress conditions. Furthermore, the reduction in large-sized tubers under severe salinity observed here agrees with Chourasia, et al.^9^, who noted that salinity not only limits yield quantity but also compromises marketable quality—a critical economic factor for potato producers.

Foliar application of branched-chain amino acids (BCAAs) mitigated the adverse effects of salinity, demonstrating their role in enhancing plant tolerance. Similar protective effects of amino acid foliar sprays have been reported by Zhang, et al.^40^ in *Camelina sativa*, Henderson, et al.^41^ in various crop species, and Sun, et al.^42^ in rice, where BCAA application improved salt tolerance through enhanced osmotic adjustment and antioxidant defense. Under non-saline conditions, the promotive effects of valine and leucine on vegetative growth and carbohydrate content observed in our study are consistent with Sun, et al.^43^, who reported that valine application enhanced carbon and nitrogen accumulation in peach trees. These amino acids may act as readily available metabolic precursors that enhance plant vigor even in the absence of stress, as suggested by Zhang, et al.^40^.

Under moderate salinity (1500 ppm), isoleucine proved particularly effective in increasing tuber number and improving the weight of large tubers. This finding aligns with Ali, et al.^44^, who reported that isoleucine plays a specific role in maintaining reproductive development and sink strength under abiotic stress conditions. The yield improvement observed in our study (approximately 40–54% over untreated controls under moderate salinity) is comparable to the range reported by Peña Calzada, et al.^2^ in soybean and by Ma, et al.^16^ in various crops following amino acid application. Ganie^26^ similarly demonstrated that BCAAs enhance osmotic adjustment and maintain physiological balance under salt stress.

Under severe salinity (3000 ppm), combined BCAA treatments—especially the V + L+I formulation—showed the greatest effectiveness in improving yield and stress tolerance. The superior performance of combined treatments highlights synergistic effects of BCAAs, where multiple metabolic pathways are simultaneously supported. This is consistent with Zeier^45^, who emphasized that combined amino acid applications can activate complementary stress-responsive pathways. The observed increases in proline, protein content, and antioxidant enzyme activities (catalase and peroxidase) in our study are key indicators of enhanced stress defense mechanisms, as similarly reported by Zulfiqar and Ashraf^46^ in their review on proline-mediated stress tolerance and by Azeem, et al.^47^ in *Moringa oleifera* under salinity.

The accumulation of proline under salinity stress observed here, particularly in combined BCAA treatments, suggests improved osmotic adjustment and protection of cellular structures. Our results are consistent with Ghosh, et al.^48^, who described proline as a multifaceted signaling molecule in plant stress responses, and with Hmidi, et al.^49^, who reported proline accumulation as an effective osmoprotective mechanism in *Cakile maritima* under salinity. Proline acts as an osmoprotectant, stabilizing proteins and membranes while scavenging reactive oxygen species (ROS), as recently reviewed by Renzetti, et al.^50^. Similarly, the increased activity of antioxidant enzymes such as catalase and peroxidase in BCAA-treated plants aligns with Hasanuzzaman, et al.^51^, who documented enhanced ROS detoxification under salinity stress. These biochemical responses confirm that BCAA application strengthens both osmotic and oxidative stress tolerance, a finding consistent with Rossi and Huang^52^ in their review on amino acid-mediated stress tolerance.

The increase in protein content under BCAA treatments observed in our study reflects improved nitrogen assimilation and metabolic activity, which are essential for growth and stress recovery. This finding agrees with Shen, et al.^24^, who reported that amino acid metabolism plays a central role in salt tolerance adaptation in *Medicago* species. The enhancement of carbohydrate content under certain treatments further supports improved photosynthetic efficiency and energy availability for tuber development, similar to results reported by Sun, et al.^42^ in peach trees.

BCAA treatments also influenced crop maturity in our study. Under moderate and severe salinity, most treatments accelerated maturity, likely as a stress-avoidance response, while the triple combination (V + L+I) extended the growth period. This suggests that combined amino acids may delay stress-induced senescence, allow longer assimilation periods and contribute to higher yield under severe conditions. A similar effect was reported by Chaichi, et al.^53^, who noted that amino acid biostimulants can modulate plant phenology under stress.

Overall, the results demonstrate that the beneficial effects of BCAAs are strongly dependent on salinity level and treatment composition. Individual amino acids were more effective under moderate stress, while combined applications were necessary to achieve optimal performance under severe stress. These findings are in line with Henderson, et al.^41^, who emphasized that biostimulant strategies should be tailored according to stress intensity. From a practical perspective, foliar BCAA application represents a promising and environmentally sustainable strategy for improving potato production in salt-affected environments.

## Conclusion

Salinity stress negatively affected potato growth and productivity under sandy soil conditions, highlighting the need for effective management strategies to improve crop tolerance in salt-affected environments. The present study demonstrated that foliar application of branched-chain amino acids (BCAAs) can effectively enhance potato tolerance to salinity stress by improving growth performance, yield attributes, and physiological resilience. Plant responses varied depending on salinity severity, with isoleucine and dual amino acid combinations showing greater effectiveness under moderate salinity, while the combined application of valine, leucine, and isoleucine provided the greatest protection under severe salinity conditions. The improved performance of treated plants was associated with enhanced osmotic adjustment and antioxidant defense mechanisms, reflected by increased proline accumulation, protein content, and antioxidant enzyme activities. From a practical perspective, this approach is relatively simple and accessible for farmers because BCAAs are water-soluble and can be applied using conventional foliar spraying equipment without requiring major modifications to existing production systems. Their use may help reduce yield losses in salt-affected areas and improve productivity under challenging conditions. However, large-scale adoption should be supported by future studies evaluating the economic feasibility and cost–benefit ratio of BCAA applications under commercial farming conditions. Overall, foliar application of BCAAs represents a promising and environmentally sustainable strategy for improving potato production in saline environments.

## Data Availability

All data available within the article.
